# A Proteomics Approach to Investigate miR-153-3p and miR-205-5p Targets in Neuroblastoma Cells

**DOI:** 10.1371/journal.pone.0143969

**Published:** 2015-12-03

**Authors:** Ketan S. Patil, Indranil Basak, Ramavati Pal, Hsin-Pin Ho, Guido Alves, Emmanuel J. Chang, Jan Petter Larsen, Simon Geir Møller

**Affiliations:** 1 Department of Biological Sciences, St. John’s University, New York, NY, 11439, United States of America; 2 Department of Chemistry, York College and the Graduate Center, The City University of New York, New York, NY, 11451, United States of America; 3 Norwegian Center for Movement Disorders, Stavanger University Hospital, 4068, Stavanger, Norway; University of S. Florida College of Medicine, UNITED STATES

## Abstract

MicroRNAs are key regulators associated with numerous diseases. In HEK293 cells, miR-153-3p and miR-205-5p down-regulate alpha-synuclein (SNCA) and Leucine-rich repeat kinase 2 (LRRK2), two key proteins involved in Parkinson’s disease (PD). We have used two-dimensional gel electrophoresis (2D-PAGE) coupled to mass spectrometry (MS) to identify a spectrum of miR-153-3p and miR-205-5p targets in neuronal SH-SY5Y cells. We overexpressed and inhibited both microRNAs in SH-SY5Y cells and through comparative proteomics profiling we quantified ~240 protein spots from each analysis. Combined, thirty-three protein spots were identified showing significant (p-value < 0.05) changes in abundance. Modulation of miR-153-3p resulted in seven up-regulated proteins and eight down-regulated proteins. miR-205 modulation resulted in twelve up-regulated proteins and six down-regulated proteins. Several of the proteins are associated with neuronal processes, including peroxiredoxin-2 and -4, cofilin-1, prefoldin 2, alpha-enolase, human nucleoside diphosphate kinase B (Nm23) and 14-3-3 protein epsilon. Many of the differentially expressed proteins are involved in diverse pathways including metabolism, neurotrophin signaling, actin cytoskeletal regulation, HIF-1 signaling and the proteasome indicating that miR-153-3p and miR-205-5p are involved in the regulation of a wide variety of biological processes in neuroblastoma cells.

## Introduction

Parkinson’s disease (PD) is the most common neurodegenerative movement disorder characterized by degeneration of dopaminergic neurons in the substantia nigra pars compacta [1]. Most PD cases are sporadic but genetic lesions in alpha-synuclein (SNCA) [2], Parkin [3], PINK1 [4], DJ-1 [5] and Leucine-rich repeat kinase 2 (LRRK2) [6] have been associated with both early- and late-onset PD. Despite extensive studies the molecular pathways leading to the onset and progression of PD are poorly understood.

MicroRNAs have been used to decode different pathways associated with several diseases [7]. However, microRNA studies within neurodegeneration are limited. In terms of PD, miR-7/miR-153 and miR-205-5p have been shown to down-regulate SNCA and LRRK2, respectively whilst DJ-1 and Parkin are regulated by miR-34b/c [8, 9, 10]. Indirect regulatory effects on PD-associated proteins have also been reported for miR-133b, miR-433, miR-184* and let-7 [11, 12, 13]. Despite limited data on microRNA regulatory pathways associated with neurodegeneration [14], microRNAs are associated with neuronal stem cell differentiation and development, synapse formation and synaptic plasticity [11, 15].

Individual microRNAs can regulate several mRNAs [16]. Therefore, comparative proteomics profiling in cells with altered microRNA levels has the potential to reveal new microRNA target proteins. The aim of this study was to combine microRNA and proteomics technologies to identify new miR-153-3p and miR-205-5p targets in neuronal SH-SY5Y cells. We selected 2D-PAGE as opposed to LC-MS as although LC-MS analysis is more comprehensive 2D-PAGE offers the possibility of identifying more subtle changes in protein abundance. Several of the protein targets identified are associated with neuronal processes and key regulatory pathways, indicating that miR-153-3p and miR-205-5p are involved in a wide variety of biological processes.

## Materials and Methods

### Cell culture and transient cell transfection

SH-SY5Y cells (CRL-2266; ATCC) were cultured in a base medium mixture (Full medium: 1:1 DMEM/Ham’s-F12) (Invitrogen) supplemented with 10% v/v fetal bovine serum (Atlanta biologics) and 2 mM GlutaMAX (Invitrogen) in 5% CO_2_ atmosphere at 37°C. Transfections were performed, in triplicate, with scrambled control mimic, miR-153-3p mimic, miR-205-5p mimic, scrambled control hairpin inhibitor, miR-153-3p hairpin inhibitor and miR-205 hairpin inhibitor, all mirVana^TM^ (Life Technologies), at a final concentration of 20 nM. Cells were seeded in 6-well plates at 5x10^5^ cells/well. 2μl μRNA (20 μM), diluted with 100 μl of Opti-MEM, and 7 μl Lipofectamine RNAiMax (Invitrogen) diluted with 100 μl of Opti-MEM was incubated for 5 minutes (min) at room temperature (RT). The two solutions were mixed and incubated for 15 min at RT. The transfection mix was diluted to 2 ml with Opti-MEM, added to the wells and incubated at 37°C for 4–6 hours before replacing with full media. Cells were harvested after 24 hours for quantitative PCR (qRT-PCR) analysis and after 48 hours for Western blotting and 2D-PAGE analysis.

### RNA isolation, RT-PCR and quantitative real-time PCR

RNA was isolated, in triplicate, 24 hours post-transfection using the miRCURY RNA isolation kit (Exiqon) treated with 1 unit/μg of RNA of DNaseI (Thermo Scientific) for 30 minutes at 37°C followed by 10 min at 65°C with 50mM EDTA. cDNA was synthesized using the qScript^TM^ microRNA cDNA Synthesis kit (Quanta Biosciences) and used for both semi-quantitative (25 cycles) and qRT-PCR. miR-153-3p forward primer (5’ GCCGGGCTTGCATAGTCACAA 3’), miR-205-5p forward primer (5’ GTTTCCTTCAT TCCACCGG 3’), U6 forward primer (5’ CGCTTCGGCAGCACATATAC 3’) and PerfeCTa^®^Universal PCR primer along with PerfeCTa^®^ SYBR^®^ GREEN SuperMix for IQ^TM^ were used for qRT-PCR in triplicates for each biological replicate.

### Western blotting

Whole cell lysates were prepared using RIPA buffer (150mM NaCl, 1% w/v NP40, 50mM Tris pH 8.0, 0.5% w/v Sodium deoxycholate, 0.1% w/v SDS) 48 hours post-transfection and used for western blot analysis following published protocol [17]. Primary antibodies used are shown in S1 Table. The secondary antibodies used were goat anti-rabbit or a goat anti-mouse secondary antibody (Jackson Immunoresearch). The reported western blot results are representative of n = 3.

### Two-dimensional gel electrophoresis

#### Sample preparation

Total protein lysates were prepared using Urea solublization buffer (7M Urea, 2M Thiourea, 4% (w/v) CHAPS and 30mM Tris, 1X protease/phosphatase inhibitor cocktail) and sonication. The supernatant of centrifuged lysates was concentrated using Amicon® Ultra centrifugal filters (10,000 MWCO). Protein concentrations were determined using the Bradford assay (Bio-Rad).

#### 2D-PAGE

Protein lysates were diluted with rehydration buffer (7 M urea, 2M thiourea, 2% (w/v) CHAPS, 40 mM DTT, 0.5% IPG buffer, pH 3–10 NL, and 0.4% Bromophenol Blue) and applied to Immobiline^TM^ DryStrip 7 cm, pH 3–10 NL (GE Healthcare) for overnight passive rehydration. Isoelectric focusing was conducted on a PROTEAN^**®**^ IEF Cell, according to the manufacturer’s recommendation (Bio-Rad). Following rehydration, proteins were reduced with DTT and subsequently alkylated with iodoacetamide in equilibration buffer (6M Urea, 2.5% SDS, 50mM Tris, pH 8.8, 20% glycerol). Electrophoresis in the second dimension was performed on 12% SDS-PAGE at 100V. Gels were stained overnight with colloidal Coomassie Blue G-250 [18].

#### Scanning and analysis of the images

Gels were scanned using EPSON scan perfection V750 PRO software (Digital ICE technologies) at 600 dpi/16-bit grayscale. ImageMaster 2D platinum 7 (GE Healthcare) was used for spot detection, background subtraction, matching, and to identify statistically significant (ANOVA) differences between protein spots i.e. fold change over control. The experiments were performed in triplicate.

#### In-gel digestion

The differentially expressed protein spots were excised, cut into small pieces and placed in 0.6 ml Eppendorf tubes. The gel pieces were destained by incubating in 200 μl of 100 mM ammonium bicarbonate: acetonitrile (50:50 v/v) with shaking. When fully destained, the gel fragments were dehydrated with two washes of 100 μl of 100% acetonitrile (ACN) and were then dried in a vacuum centrifuge (Speed-Vac) for 5 min. The proteins were then cleaved enzymatically into peptides. For this, trypsin solution (2 μl of 0.02 μg/μl) was added to the wet the gel pieces and incubation was carried out for 4 hours at RT. Thirty μl of 50 mM ammonium bicarbonate was added to the gel pieces and left overnight at RT to allow for diffusion of the peptides from the gel. The digested proteins were stored at -80°C until further analysis.

#### Peptide mass fingerprinting

After digestion, POROS 20 R2 resin (Applied Biosystems) was added to the digested gel samples with 5% formic acid and 0.2% trifluoroacetic acid for extraction at 4°C for 4 hours on a shaker. Prior to MALDI-MS analysis, the peptide digests were further desalted using ZipTip C_18_ (Millipore). The ZipTips were conditioned with 10 μl of 0.1% TFA twice, 70% ACN/0.1% TFA twice, and 10 μl of 0.1% TFA twice. Samples containing the digest and bead mixture were transferred to the ZipTips and bound to the C18 resin. The loaded tips were then washed with 10 μl of 0.1% TFA. The peptide digests were eluted by placing 2 μl of 10 mg/mL CHCA matrix solution in 0.003% TFA, 13% ethanol, and 84% ACN onto the top of the ZipTips and slowly dispensing onto the MALDI plate. Mass spectrometric analysis was performed using a Thermo LTQ XL linear ion trap mass spectrometer (Thermo Scientific) equipped with a vacuum MALDI source, after the solvent evaporated at RT and the CHCA matrix was crystallized with peptides on the MALDI plate. A data-dependent acquisition was performed using Xcalibur software, in which the top 40 of the most abundant precursor ions from the survey scan (mass range 700–3500 Da) were chosen and MS/MS acquisition was triggered to fragment them by CID (collision-induced dissociation). The normalized collision energy was 50%, and the isolation width was 3 Da. The raw-files from the LTQ mass spectrometer were analyzed by using Mascot Distiller 2.3.2 (Matrix Science, Boston, MA) for protein identification. Peptide masses were matched against the taxonomy *Homo sapiens* in the National Centre for Biotechnology Information non-redundant (NCBInr) database. One missed trypsin cleavage per peptide was allowed and an initial mass tolerance of 0.3 Da was used in all searches. Complete carboxyamidation of cysteine sulfhydryls and partial oxidation of methionine were assumed [19].

### Cell viability and reactive oxygen species measurements

Cell viability was measured using the neutral red uptake assay 48 hours post-transfection. Cells were washed with PBS, 100μl of neutral red working solution (40μg/ml) added to each well and plates were incubated for 2 hours. Cells were then washed with PBS, neutral red extracted using 150μl of destain solution (50% ethanol, 1% glacial acetic acid, 49% deionized water) per well and the plates were subjected to shaking for 10 min. Absorbance was measured at 540nm using an Epoch microplate spectrophotometer (BioTeck).

Cellular reactive oxygen species (ROS) were measured using 2’, 7’- dichlorofluoresceine diacetate (DCF-DA) (Sigma-Aldrich). Cells were plated on solid black opaque plates at 5x10^4^ cells per well and after 48 hours incubated with 100μl of DCF-DA (25μM) for 45 min. Fluorescence was measured using a GLoMas®-Multi Detection System fluorescence plate reader (Promega) at 485nm (excitation) and 528nm (emission). The assays were performed in triplicate.

### Image analysis, statistical analysis and contextual analysis

Western blot images were analyzed using IQTL software (GE Healthcare). Microsoft excel tools was used for two-tailed Student’s t-test. The standard error was used to display variation. The targets of miR-153-3p and miR-205-5p were used as input queries for the Partek Genomics Suite software, version 6.6 (Partek) to perform Gene ontology (GO) analysis and generate interactive maps and pathways.

## Results and Discussion

### Overexpression and inhibition of miR-153-3p and miR-205-5p in SH-SY5Y cells

**Fig 1:**
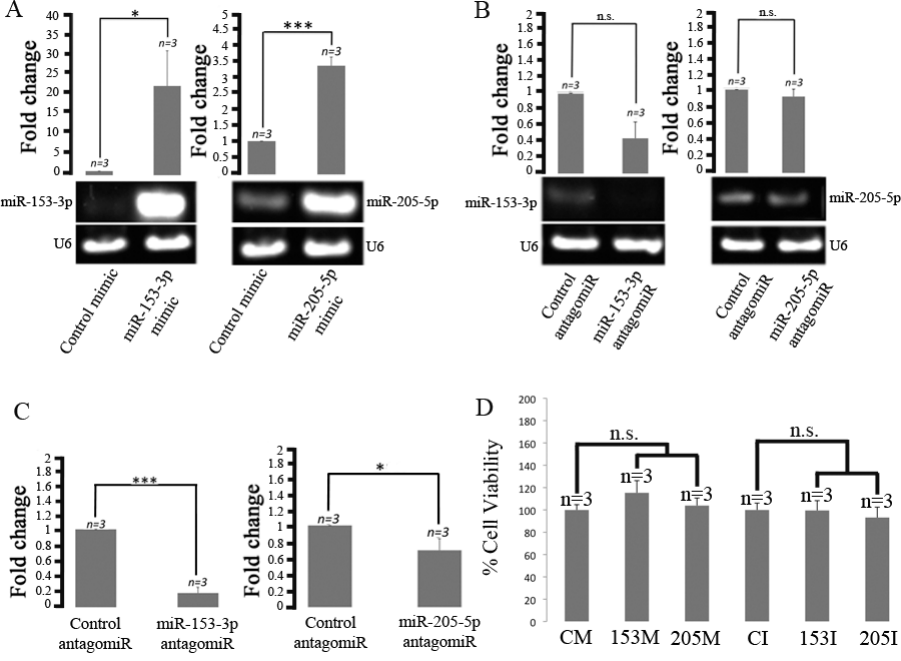
Overexpression and inhibition of miR-153-3p and miR-205-5p and their effect on cell viability in SH-SY5Y cells. (A-B) Semi-quantitative RT-PCR analysis showing miR-153-3p expression levels in response to mimic (A) and antagomir (B) transfections. miR-205-5p expression levels in response to mimic (A) and antagomir (B) transfections. (C) qRT-PCR analysis confirming the antagomir mediated inhibition of miR-153-3p (C) and miR-205-5p. (D) Cell viability assay. U6 was used as loading controls for qRT-PCR. Error bars indicate SEM (n = 3); *, *p* < 0.05, ***; *p* < 0.001.

miR-153 overexpression in HEK293 cells downregulate SNCA whilst miR-205 overexpression in HEK293 cells has been shown to downregulate LRRK2 [8, 9]. In this study, we selected the neuroblastoma cell line SH-SY5Y as its neuronal characteristics represents a better platform to dissect microRNA-regulated pathways and mechanisms associated with PD. miR-153-3p was successfully overexpressed using miR-153-3p mimic and inhibited using miR-153-3p antagomir in SH-SY5Y cells (Fig 1A and 1B). Similarly, miR-205-5p was successfully overexpressed using miR-205-5p mimic and inhibited using miR-205-5p antagomir (Fig 1A and 1B). Further, we used qPCR to verify the down regulation of both the microRNAs due to the antagomirs (Fig 1C).

### Altered levels of miR-153-3p and miR-205-5p results in protein changes associated with a spectrum of biological processes

We next sought to identify additional targets of miR-153-3p and miR-205-5p in SH-SY5Y cells using 2D-PAGE analysis. However, before performing 2D-PAGE analysis we showed that miR-153-3p and miR-205-5p transfections had no significant effect on SH-SY5Y cell viability ensuring that any observed protein changes were due to changes in miR-153-3p and miR-205-5p levels (Fig 1D).

**Fig 2:**
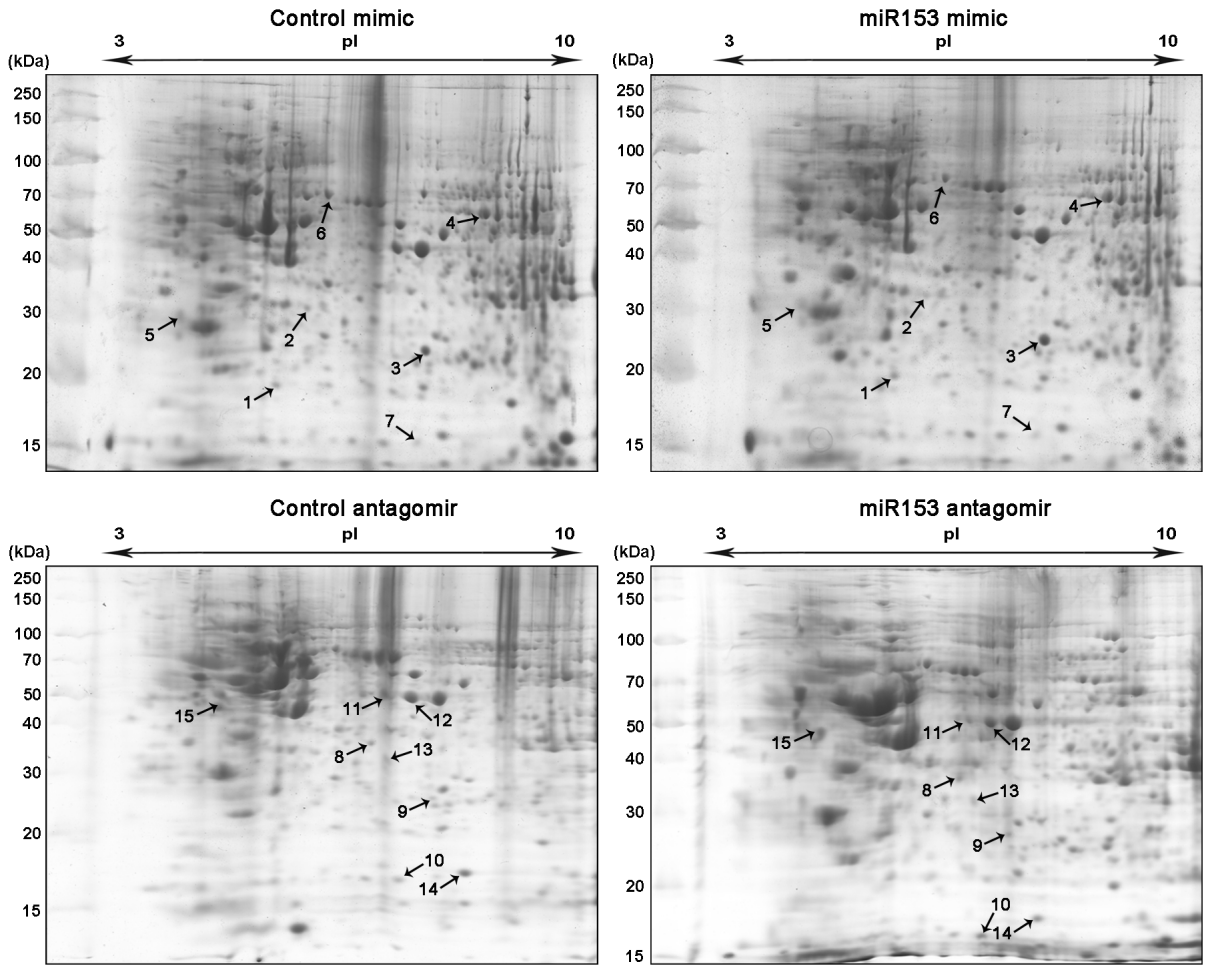
Comparative proteomic profiling in SH-SY5Y. Two-dimensional gels of control mimic, miR-153-3p mimic, control antagomir and miR-153-3p antagomir transfected cells. n = 3 for all experiments. Numbers (1–15) represent differentially expressed protein spots identified by MS, reported in Table 1.

**Fig 3:**
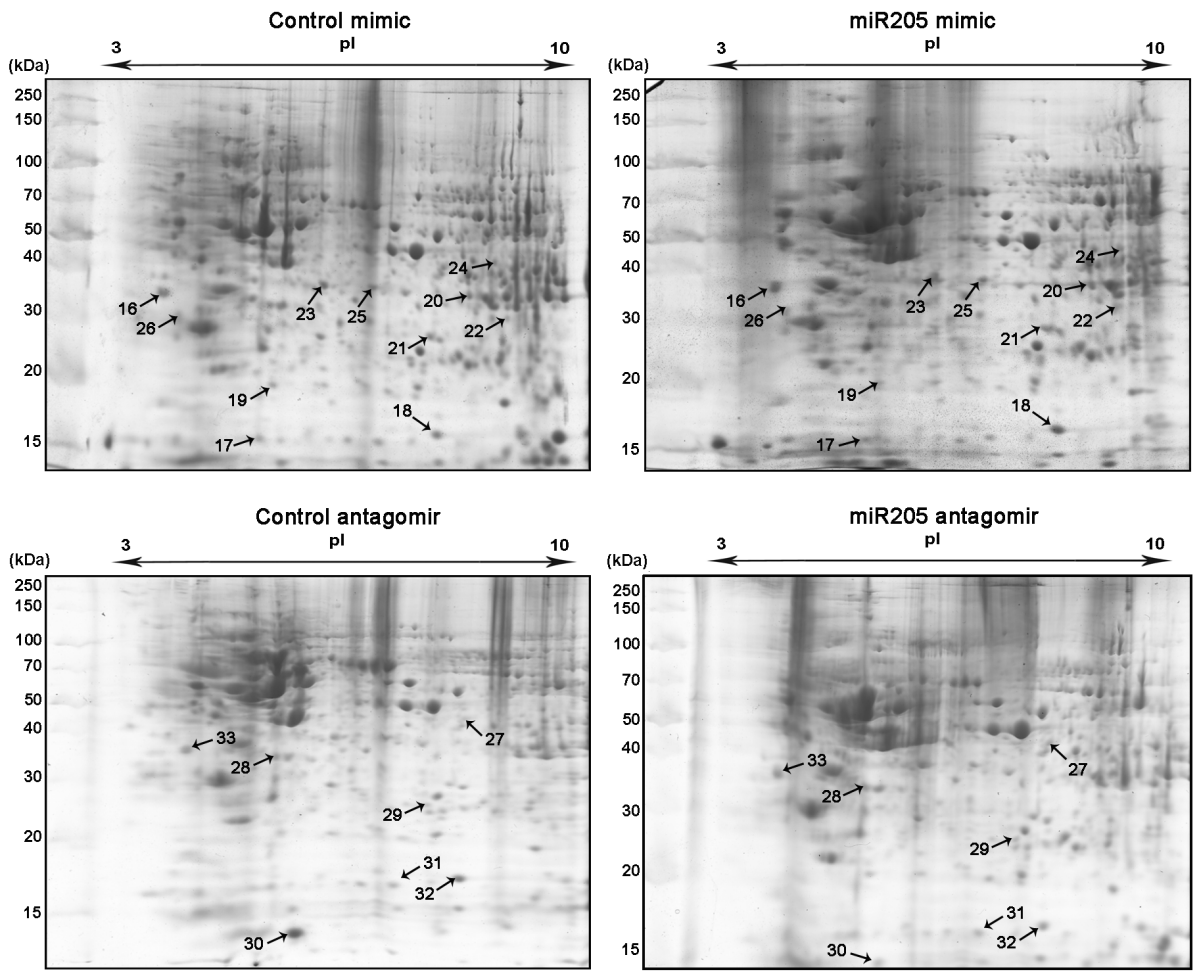
Comparative proteomic profiling in SH-SY5Y. Two-dimensional gels of control mimic, miR-205-5p mimic, control antagomir and miR-205-5p antagomir transfected cells. n = 3 for all experiments. Numbers (16–33) represent differentially expressed protein spots identified by MS, reported in Table 1.

We performed comparative 2D-PAGE analysis comparing control mimic and control antagomir transfected cells with cells transfected with the miR-153-3p mimic and the miR-153-3p antagomir, respectively (Fig 2). The same comparative analyses were performed for SH-SY5Y cells transfected with the miR-205-5p mimic and the miR-205-5p antagomir (Fig 3).

We identified thirty-three protein spots that showed significant abundance changes (fold change > 1.4, n = 3, p-value < 0.05) between control transfected and miR-153-3p/miR-205-5p-transfected SH-SY5Y cells. In response to altered levels of miR-153-3p seven protein spots were up-regulated whilst eight protein spots were down-regulated (Fig 2, Table 1). In response to miR-205-5p perturbations twelve protein spots were up-regulated whilst six protein spots were down-regulated (Fig 3, Table 1). The protein spots were subjected to MS and the fragment spectra were searched against the NCBInr database (taxonomy *Homo sapiens*) using Mascot Distiller revealing the identity of the differentially expressed proteins (S1 Fig; Table 1, S2 Table).

**Table 1:** List of differentially regulated proteins in SH-SY5Y cells in response to mimics and antagomirs of miR-153-3p and miR-205-5p.

Spot No.	UniProt Accession	Description	Sequence coverage	Fold change	Significance p-value
**miR-153-3p mimic—upregulated proteins**					
1	P32119	Peroxiredoxin-2	27	1.66 ± 0.13	0.03
2	P11177	Pyruvate dehydrogenase beta subunit	12	2.26 ± 0.19	0.04
3	P09429	High mobility group protein B1 (HMGB-1)	26	1.64 ± 0.11	0.02
4	P25786	Proteasome subunit alpha type-1 isoform 2	20	1.49 ± 0.09	0.03
	P31948	Stress-induced-phosphoprotein 1	13		
**miR-153-3p mimic—downregulated proteins**					
5	Q8IW75 ^***x***^	Serpin A12 precursor	1	1.44 ± 0.04	0.04
6	P38646	Heat shock 70kDa protein 9 (mortalin)	24	1.84 ± 0.22	0.003
7	Q9UHV9	Prefoldin subunit 2	16	2.34 ± 0.25	0.04
**miR-153-3p antagomir—Upregulated proteins**					
8	P05388	60S acidic ribosomal protein P0	35	2.24 ± 0.13	0.05
9	Q13162	Peroxiredoxin-4 precursor	30	2.03 ± 0.43	0.05
	Q14CN2	Ca2+-activated chloride channel protein 2	3		
10	P22392	Human Nucleoside Diphosphate Kinase B (Nm23)	36	1.61 ± 0.14	0.008
**miR-153-3p antagomir—downregulated proteins**					
11	P06733	Alpha-enolase	13	3.18 ± 0.09	0.01
12	P13929	Beta-enolase	22	1.84 ± 0.13	0.03
13	Q9UBR2	Cathepsin Z precursor	6	2.65 ± 0.16	0.01
14	P23528	Cofilin-1	53	2.11 ± 0.20	0.004
15	P68104	Elongation factor 1-alpha 1	10	2.55 ± 0.64	0.01
	P62258	14-3-3 protein epsilon	24		
**miR-205-5p mimic—upregulated proteins**					
16	Q13765	Nascent-polypeptide-associated complex alpha (HSD48)	25	1.80 ± 0.28	0.05
17	P63241	Eukaryotic translation initiation factor 5A-1 isoform B	22	2.28 ± 0.27	0.05
18	P23528	Cofilin-1	43	2.11 ± 0.77	0.009
19	P32119	Peroxiredoxin-2	21	1.99 ± 0.03	0.05
20	P04083	Annexin A1	7	2.08 ± 0.16	0.04
21	P25786	Proteasome subunit alpha type-1 isoform 2	9	1.61 ± 0.16	0.001
22	Q13126	Methylthioadenosine phosphorylase	16	1.80 ± 0.24	0.04
23	Q13347	Eukaryotic translation initiation factor 3 subunit I	13	1.55 ± 0.03	0.04
24	P62333 ^**y**^	Proteasome subunit p42	3	1.90 ± 0.09	0.04
**miR-205-5p mimic—downregulated proteins**					
25	P50213	Isocitrate dehydrogenase [NAD] subunit alpha, mitochondrial precursor	23	2.02 ± 0.24	0.007
	P04406 ^***z***^	Glyceraldehyde-3-phosphate dehydrogenase	4		
26	Q8IW75 ^***x***^	Serpin A12 precursor	1	2.38 ± 0.50	0.03
**miR-205-5p antagomir—upregulated proteins**					
27	Q13148	TAR DNA-binding protein 43	7	2.03 ± 0.09	0.01
28	Q07955	Serine/arginine-rich splicing factor 1 isoform 1	53	2.24 ± 0.58	0.04
29	Q13162	Peroxiredoxin-4 precursor	33	1.66 ± 0.09	0.04
**miR-205-5p antagomir—downregulated proteins**					
30	P09382	Human Galectin-1	52	1.80 ± 0.22	0.03
31	P22392	Human Nucleoside Diphosphate Kinase B (Nm23)	24	1.55 ± 0.00	0.03
32	P23528	Cofilin-1	34	2.11 ± 0.30	0.02
33	Q13765	Nascent-polypeptide-associated complex alpha (HSD48)	25	1.69 ± 0.00	0.05
	Q01105 ^***x***^	Protein SET	5		

^**x, y, z**^–Single peptides identified from individual protein spots in two (^**x**^), three (^**y**^) or seven (^**z**^) independent MALDI-MS detections. All the protein spots were picked and analyzed from at-least two independent experiments. Fold change ± error are calculated with respect to control mimic and control inhibitor by ImageMaster 2D platinum 7 (GE) software. The significance was calculated using two-tailed *t*-test.

### Regulation of key neuronal processes by miR-153-3p and miR-205-5p

Overexpression of miR-153-3p resulted in up-regulation of proteasome subunit alpha type-1 isoform 2 (PSMA1) (Table 1, spot 4; S2 Fig). miR-205-5p overexpression also increased the abundance of proteasome subunit p42 (PSMC6) (Table 1, spot 24; S2 Fig) and proteasome subunit alpha type-1 isoform 2 (PSMA1) (Table 1, spot 21). Efficient proteasome activity is vital in neurons as inappropriate degradation of misfolded proteins, such as amyloids and SNCA, results in aggregate formation, a hallmark of AD and PD [12, 20].

miR-153-3p overexpression also resulted in increase abundance of Prefolding subunit 2 (PFDN2) (Table 1, spot 7), which transfers misfolded proteins to chaperonin ensuring proper folding [21]. This indicates that miR-153-3p may up-regulate PFDN2 in response to increased levels of misfolded proteins as a neuroprotective mechanism.

We also found that cathepsin Z (CTSZ) (Table 1, spot 13) is down-regulated in response to miR-153-3p inhibition. In aging mouse brains cathepsin is upregulated, impairing neuronal survival and neuritogenesis, indicating that miR-153-3p may regulate cathepsin levels to maintain a healthy neuronal population [22].

Inhibition of miR-153-3p also results in the up-regulation of the calcium activated chloride channel family member 4 (CLCA4) (Table 1, spot 9). Calcium activated chloride channels are highly expressed in microglia and activated microglia and a reduction in toxicity is seen in response to CLCA inhibitors [23]. CLCA4 inhibition by miR-153-3p may contribute to neuroprotection.

**Fig 4:**
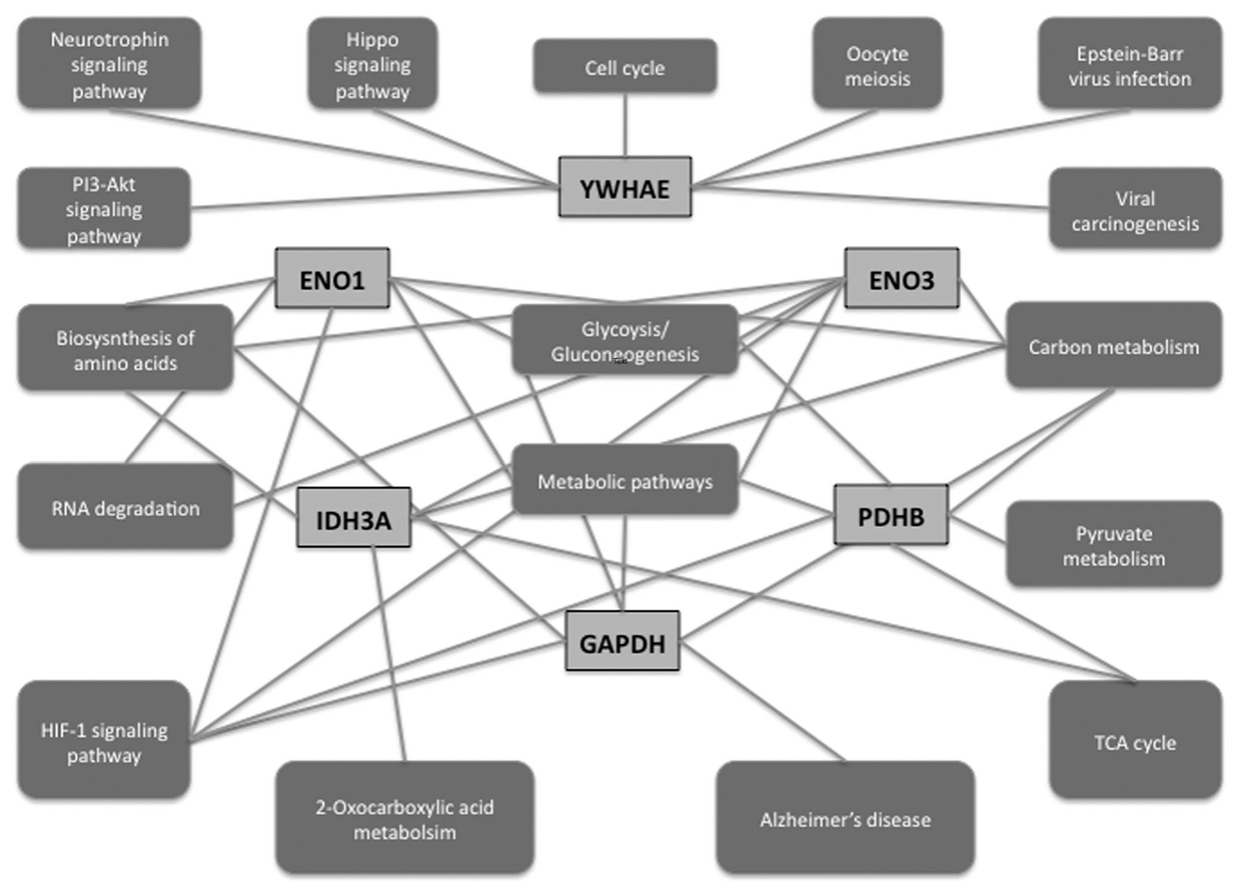
Protein association network showing interconnecting relationships between miR-153-3p and miR-205-5p target proteins through key regulatory pathways. YWHAE: 14-3-3 epsilon protein; ENO1: Alpha-enolase; ENO3: beta-enolase; IDH3A: Isocitrate dehydrogenase [NAD] subunit alpha; PDHB: Pyruvate dehydrogenase complex beta subunit; GAPDH: Glyceraldehyde-3-phosphate dehydrogenase.

The stress-induced phosphoprotein 1 (STIP1) (Table 1, spot 4) is also up-regulated in response to the miR-153-3p mimic whilst the expression of mortalin (Heat shock 70kDa protein 9-HSPA9) (Table 1, spot 6) is down-regulated. The STIP1 protein forms a complex with HSC70 and HSP90 [24] and STIP1 is elevated in serum from patients with neuro-Behçet's disease [25]. We also found that 14-3-3 protein epsilon (14-3-3E) (Table 1, spot 15), involved in cell cycle regulation, PI3-Akt signaling, Hippo signaling, Neurotrophin signaling, and viral carcinogenesis (Fig 4), is down-regulated in response to miR-153-3p inhibition [26]. Several 14-3-3 isoforms are present in Lewy bodies suggesting the involvement of 14-3-3 proteins in neurodegeneration [27]. The regulation of 14-3-3 proteins by microRNAs has been documented where 14-3-3zeta is a direct target of miR-451 [28].

**Fig 5:**
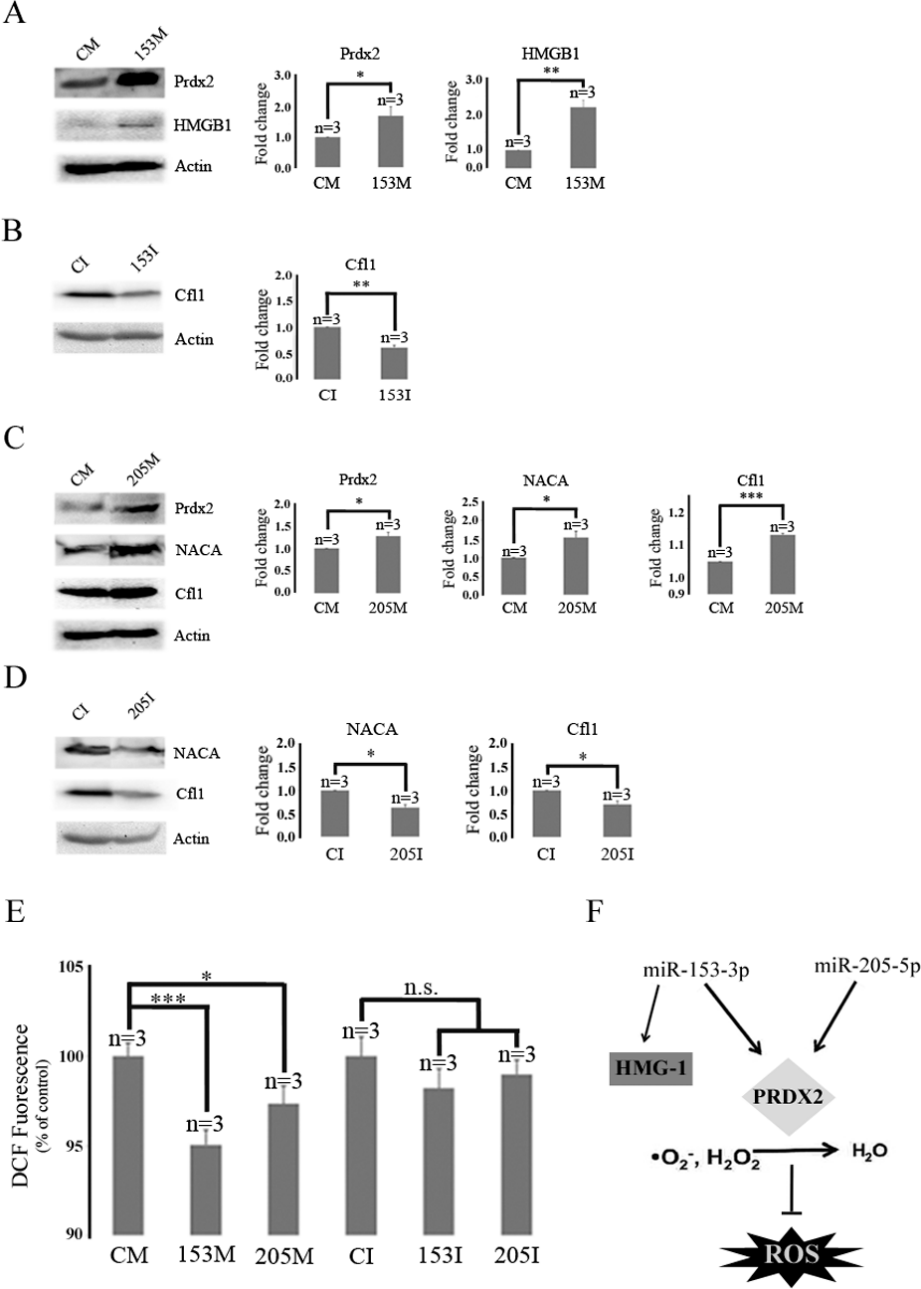
Verification of differentially expressed proteins by Western blot analysis and ROS changes in response to miR-153-3p and miR-205-5p. Western blot analysis showing effect of miR-153-3p mimic on (A) PRDX2 and HMGB1 levels (B) effect of miR-153-3p antagomir on Cfl1 levels, (C) effect of miR-205-5p mimic on PRDX2, NACA and Cfl1 levels, (D) effect of miR-205-5p antagomir on NACA and Cfl1 levels. (E) Quantification of ROS levels in SH-SY5Y cells by modulating miR-153-3p and miR-205-5p levels. Percentage change in DCF fluorescence compared to control is shown. (F) Proposed pathway for ROS reduction due to miR-153-3p and miR-205-5p by regulation of PRDX2. Error bars indicate SEM (n = 3); *, *p* < 0.05; **, *p* < 0.01, ***; *p* < 0.001.

In response to miR-153-3p inhibition cofilin-1 was down-regulated (Table 1, spot 32), verified by western blot analysis (Fig 5B), whilst miR-205-5p overexpression resulted in cofilin-1 up-regulation (Table 1, spot 18). Cofilin-1 is involved in protein translocation, rod-shaped actin bundle formation and is activated by amyloid-beta (Abeta 1–42) [29, 30]. Rod-shaped actin bundles are sites for amyloid-precursor protein accumulation in AD [31]. Western blot analysis confirmed cofilin-1 regulation by the miR-205-5p mimic (Fig 5C) and the antagomir (Fig 5D).

Our data indicates that both miR-205-5p and miR-153-3p influence direct and peripheral processes associated with neurodegenerative disorders, providing clues towards the possible regulation of key pathways (S4 Table, Fig 4).

#### Peroxiredoxins are regulated by both miR-153-3p and miR205-5p

We found that miR-153-3p overexpression leads to an up-regulation of peroxiredoxin 2 (PRDX2) (Table 1, spot 1) whilst miR-153-3p inhibition results in peroxiredoxin-4 (PRDX4) precursor up-regulation (Table 1, spot 9). Similar effects were also observed for miR-205-5p (Table 1, spot 19 & 29). The PRDX family protects cells from oxidative stress-induced apoptosis and have been associated with neurodegeneration [32]. PRDX2 overexpression in MN9D neuronal cells results in a ROS decrease and prevention of 6-OHDA-induced ASK1 activation by regulating the redox status of thioredoxin (Trx), whilst PRDX2 knockdown causes a ROS increase [33]. Cytosolic PRDX2 (S3 Table) can also act as a chaperone protecting citrate synthase, insulin and SNCA from stress-induced aggregation [34, 35]. We verified the miR-153-3p- and miR-205-5p-mediated increase in PRDX2 by western blot analysis (Fig 5A and 5C). PRDX4 is putative tumor driver where down-regulation of PRDX4 in glioblastoma multiformes (GBMs) results in decreased cell growth and increased levels of ROS, DNA damage, and apoptosis [36].

The regulation of PRDXs by miR-153-3p and miR-205-5p suggest that miR-153-3p and miR-205-5p may affect cellular ROS levels. Indeed, overexpression of miR-153-3p and miR-205-5p causes significant ROS reduction (Fig 5E). Combined this indicate that miR-153-3p and miR205-5p influence PRDX levels, which may affect ROS levels (Fig 5F).

### miR-153-3p and miR-205-5p alter known cell cycle regulators

Numerous microRNAs are involved in the cell cycle, cancer proliferation and metastasis [37]. In response to miR-153-3p inhibition we identified Nucleoside diphosphate Kinase B (Nm23) (Table 1, spot 10) and tumor suppressor alpha-enolase (Table 1, spot 11), two cell cycle regulatory proteins [38, 39]. miR-153-3p inhibition results in increased abundance of Nm23 (Fig 2, spot 10), known as a transcriptional activator of *c-myc* [40]. In contrast, alpha-enolase is down-regulated in response to miR-153-3p inhibition (Table 1). Interestingly, alpha-enolase can bind to the *c-myc* promoter, but in contrast to Nm23, represses *c-myc* expression [41].

We also found that altered levels of miR-205-5p affect proteins associated with tumor proliferation and invasion (Table 1). miR-205-5p inhibition down-regulates Nm23 (Table 1, spot 31) and Protein SET (Fig 2, spot 33). Protein SET, part of the inhibitor of acetyltransferases (INHAT) complex, is up-regulated in numerous tumors [42]. Interestingly, a jun co-activator, Nascent-polypeptide-associated complex alpha (HSD48), was up-regulated (Table 1, spot 16) by miR-205-5p overexpression whilst its expression decreased (Table 1, spot 33) by miR-205-5p inhibition [43, 44]. HSD48 (NACA) regulation by miR-205-5p was confirmed by western blot analysis (Figs 5C and 4D).

Galectin-1, a beta-galactoside binding protein associated with cell proliferation and differentiation is also down-regulated in response to miR-205-5p inhibition (Table 1, spot 30) [45].

Combined these results indicate that miR-153-3p and miR-205-5p may play a role in cell proliferation and migration involving various target proteins.

### miR-153-3p and miR-205-5p have roles in regulating proteins involved in metabolic pathways

Glucose stimulation increases miR-153 expression and miR-153 expression is reduced in PTPRN2 (Protein tyrosine phosphatase receptor type N polypeptide 2) mouse knockout models [46]. We found that the expression of adipokine Serpin A12 (SERPINA12) (Table 1, spot 5) is down-regulated in response to miR-153-3p overexpression whilst the pyruvate dehydrogenase complex beta subunit (PDHB) (Table 1, spot 2), a key enzyme linking the glycolytic pathway to the TCA cycle, is up-regulated (Table 1) [47].

miR-153-3p overexpression also resulted in the up-regulation of High mobility group protein B1 (HMGB1) (Table 1, spot 3), involved in remodeling chromatin affecting gene expression (S3 Table) [48]. HMGB1-deficient mice have lethal hypoglycemia causing death within 24 hours [49]. To verify the up-regulation of HMGB1 in response to miR-153-3p overexpression we performed western blot analysis (Fig 5A). Interestingly, cofilin-1 (CFL1) (Table 1, spot 14), which decreases in abundance as a result of miR-153-3p inhibition, is shown to act as glucocorticoid receptor inhibitor [50].

Similar to miR-153-3p, miR-205-5p also down-regulates Serpin A12 (Table 1, spot 26). Furthermore, miR-205-5p down-regulates isocitrate dehydrogenase [NAD] subunit alpha (IDH3A) (Table 1, spot 25), a key enzyme in the TCA cycle and GAPDH (Table 1, spot 25)[51, 52]. miR-205-5p also up-regulates Annexin A1 (Table 1, spot 20), a protein that regulates phospholipase A_2_ activity [53].

Collectively, miR-153-3p and miR-205-5p appears to regulate proteins involved in metabolic pathways and in particular carbohydrate metabolism (S4 Table).

### miR-205-5p is associated with transcriptional regulation

miR-205-5p appears to be affecting the abundance of proteins that influence mRNA expression and processing (Table 1 and Fig 2). The serine/arginine-rich splicing factor 1 (SRSF-1) (Table 1, spot 28), which ensures splicing accuracy and regulates alternative splicing, is up-regulated in response to miR-205-5p inhibition [54]. Indeed, HSD48 (Table 1, spot 16 & 33), which is regulated by miR-205-5p, is a transcription regulator [55]. miR-205-5p inhibition also causes increased abundance of the TAR DNA-binding protein 43 (TDP-43) (Table 1, S3 Table, spot 27) that promotes CFTR exon skipping and regulates transcription [56]. Nm23, a gene expression modulator, is also regulated by miR-205-5p showing decreased levels in response to miR-205-5p inhibition (Table 1, spot 31) [57].

As microRNAs are most commonly involved in translational regulation, the up-regulation of eukaryotic translation initiation factor 5A-1 isoform B (EIF5A) (Table 1, spot 17) and (EIF3I) (Table 1, spot 23), in response to miR-205-5p was not surprising.

### Concluding remarks

MicroRNA biology is complex and we have shown that miR-153-3p and miR-205-5p influences the abundance of numerous proteins integral to many biological processes in neuroblastoma cells (Fig 4, S3 Table). Interestingly, we observed that some proteins (cofilin-1 and HSFD48) show reciprocal regulatory effects in response to miRNA mimic and antagomir whilst other proteins did not show this reciprocal regulation. This suggests that the proteins identified in this study represent a combination of direct and indirect targets of miR-153-3p and miR-205-5p.

Some of these processes associated with the identified proteins are fundamental in nature whilst others are specifically associated with cell survival, cell proliferation and neuroprotection. Although we acknowledge that the altered abundance of a small number of proteins in a pathway may not necessarily indicate that the entire pathway is affected, our study highlights that to fully understand microRNA-mediated processes a holistic approach is needed, which will pave the way for further insight into neuronal processes associated with normal development and disease.

## Supporting Information

S1 Fig MS/MS annotated spectra of the proteins identified with single peptide for (A) spot 5 (Q81W75^*x*^), Serpin A12 precursor (Mascot score: 32, score > 16 indicates homology, expect 0.0072); (B) spot 33 (Q01105^*x*^), Protein SET (Mascot score: 58, score > 37 indicates identity, expect 6.2e^-05^); (C) spot 24 (P62333^*y*^), Proteasome subunit p42 (Mascot score: 19, score > 18 indicates homology, expect 0.05); (D) spot 25 (P04406^*z*^), Glyceraldehyde-3-phosphate dehydrogenase (Mascot score: 67, score > 38 indicates identity, expect 2.6e^-05^). Shown are representative spectra for the peptide sequence shown at the top of each spectrum. The spectra are derived from Mascot search results.
(TIF)

S2 Fig Molecular map of the 26S proteosome showing proteins involved in ubiquitin mediated proteolysis. Proteasome subunit alpha type-1 isoform 2 (PSMA1) (regulated by miR-153-3p and miR-205-5p) and proteasome subunit p42 (PSMC6) (regulated by miR-205-5p) are integral parts of the 26S proteosome.
(TIF)

S1 Table List of primary antibodies used in this study. (DOCX)

S2 Table Details of identified proteins by mass spectrometry. (XLSX)

S3 Table GO annotation of all proteins identified showing molecular function and cellular location. (DOCX)

S4 Table Cellular processes and pathway analysis of the differentially regulated proteins in response to mimics and antagomirs of miR-153-3p and miR-205-5p. (DOCX)
