# Supplementary figures and images for: A Proteomics Approach to Investigate miR-153-3p and miR-205-5p Targets in Neuroblastoma Cells
Source: PLoS One. 2015 Dec 3;10(12):e0143969. doi: 10.1371/journal.pone.0143969 (PMC4669106; doi:10.1371/journal.pone.0143969)

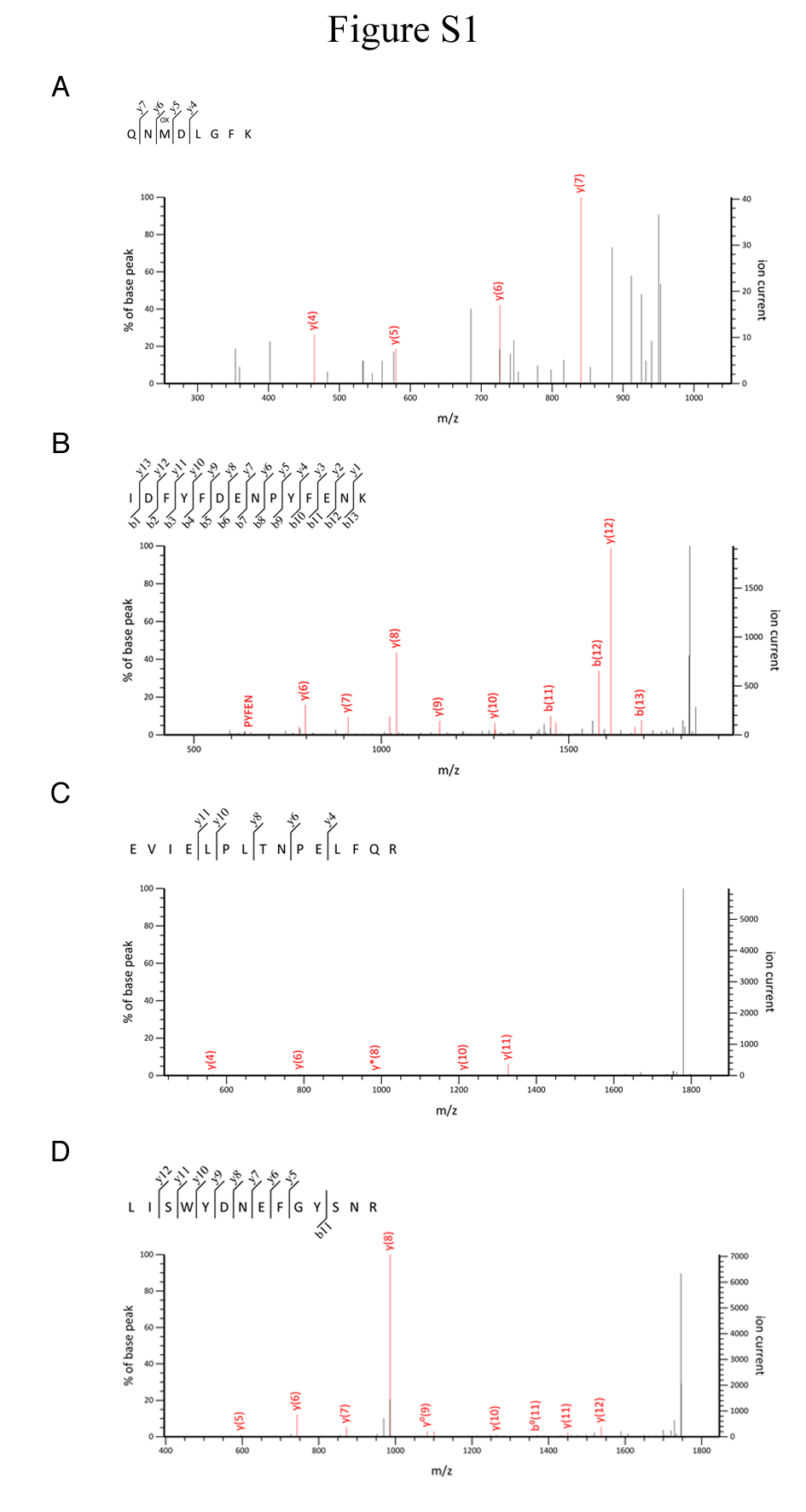

Supplement: S1 Fig — Shown are representative spectra for the peptide sequence shown at the top of each spectrum. The spectra are derived from Mascot search results. (TIF) [file pone.0143969.s001.tif]

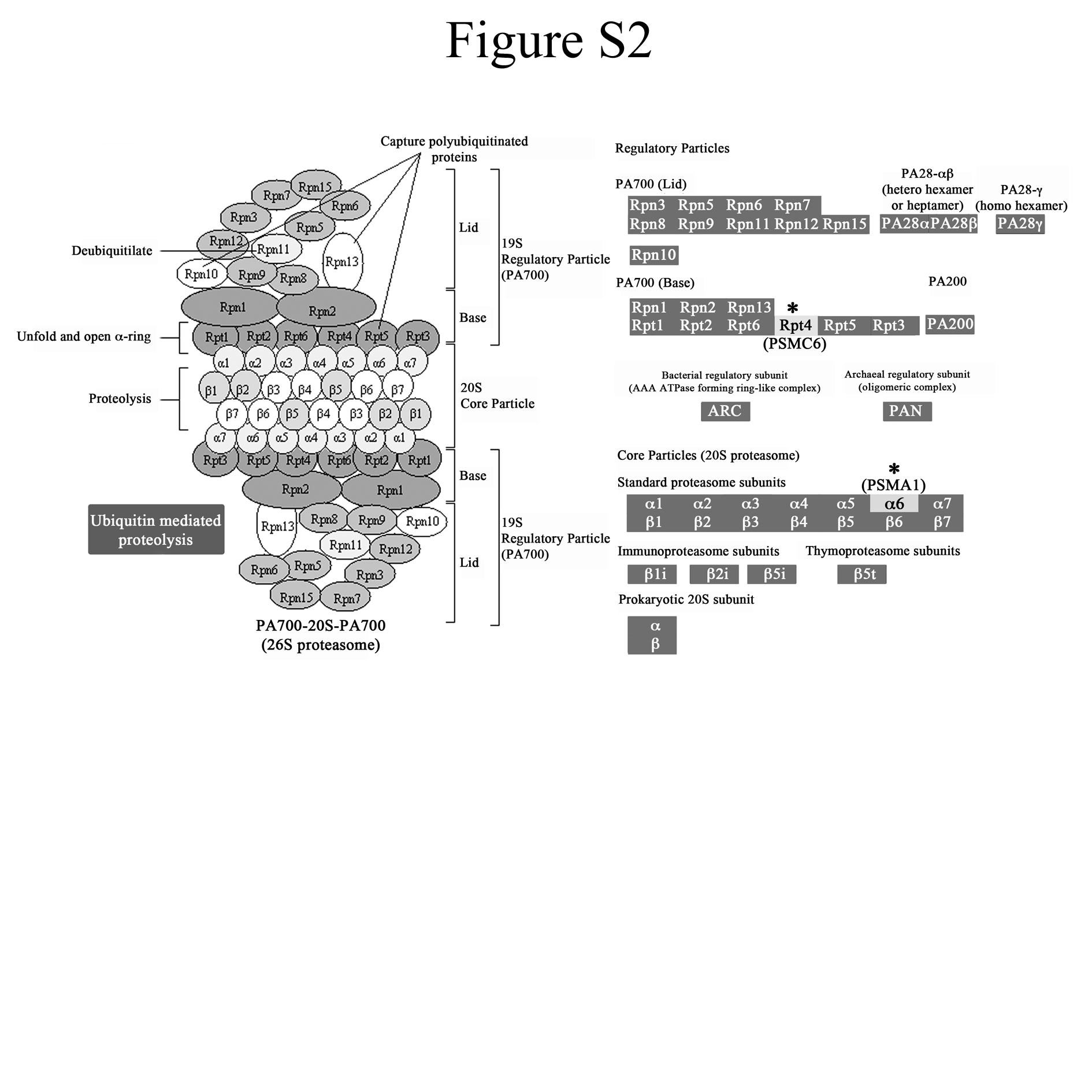

Supplement: S2 Fig — Proteasome subunit alpha type-1 isoform 2 (PSMA1) (regulated by miR-153-3p and miR-205-5p) and proteasome subunit p42 (PSMC6) (regulated by miR-205-5p) are integral parts of the 26S proteosome. (TIF) [file pone.0143969.s002.tif]
