# Supplementary material for: A Proteomics Approach to Investigate miR-153-3p and miR-205-5p Targets in Neuroblastoma Cells
Source: PLoS One. 2015 Dec 3;10(12):e0143969. doi: 10.1371/journal.pone.0143969 (PMC4669106; doi:10.1371/journal.pone.0143969)
Supplement: S1 Table — (DOCX) [file pone.0143969.s003.docx]

**Table S1. List of primary antibodies used in this study**

| **Antibody** | **Source** |
| --- | --- |
| Rabbit polyclonal anti-SNCA antibody | Abcam, Cambridge, MA |
| Rabbit polyclonal anti-LRRK2 antibody | Abcam, Cambridge, MA |
| Mouse monoclonal anti-β-Actin antibody | Sigma-Aldrich, St. Louis, MO |
| Rabbit polyclonal anti-HMGB1 antibody | Santa Cruz, Dallas, TX |
| Rabbit polyclonal anti-PRDX2 antibody | Dr. Katja Becker, Justus-Liebig University Giessen, Germany |
| Rabbit polyclonal anti-CFL1 antibody | Dr. Cristina Fernandez-Valle​, University of Central Florida, USA |
| Rabbit polyclonal anti-NACA antibody | Dr. Susanne Hradetzky, Hannover Medical School, Germany |
