# Supplementary material for: A Proteomics Approach to Investigate miR-153-3p and miR-205-5p Targets in Neuroblastoma Cells
Source: PLoS One. 2015 Dec 3;10(12):e0143969. doi: 10.1371/journal.pone.0143969 (PMC4669106; doi:10.1371/journal.pone.0143969)
Supplement: S3 Table — (DOCX) [file pone.0143969.s005.docx]

**Table S3. GO annotation of all proteins identified showing molecular function and cellular location**

| **Spot Description Gene Molecular Function Location**  **No. Symbol** | | | | |
| --- | --- | --- | --- | --- |
| 1,19 | Peroxiredoxin-2 | PRDX2 | Antioxidant activity, oxidoreductase activity, thioredoxin peroxidase activity. | Cytosol |
| 2 | Pyruvate dehydrogenase beta subunit | PDHB | Pyruvate dehydrogenase (acetyl-transferring) activity. | Mitochondrial matrix |
| 3 | High mobility group protein B1 (HMGB-1) | HMGB1 | DNA binding, bending, RAGE receptor binding, chemo-attractant activity, cytokine activity, damaged DNA binding, sequence-specific DNA binding transcription factor activity. | Nucleus, Chromosome |
| 4,21  4 | Proteasome subunit alpha type-1 isoform 2 | PSMA1 | RNA binding, lipopolysaccharide binding, threonine-type endopeptidase activity. | Cytoplasm, Nucleus, |
|  | Stress-induced-phosphoprotein 1 | STIP1 |  | Cytoplasm, Nucleus |
| 5,26 | Serpin A12 precursor | SERPINA12 | Serine-type endopeptidase inhibitor activity | Secreted |
| 6 | Heat shock 70kDa protein 9 (mortalin) | HSPA9 | ATP binding, unfolded protein binding | Mitochondria, Nucleus, |
| 7 | Prefoldin subunit 2 | PFDN2 | Unfolded protein binding | Nucleus, Cytoplasm, Mitochondrion |
| 8 | 60S acidic ribosomal protein P0 | RPLP0 | RNA binding, constituent of ribosome | Nucleus, Cytoplasm |
| 9,29  9 | Peroxiredoxin-4 precursor | PRDX4 | Thioredoxin peroxidase activity | Cytoplasm, Secreted |
|  | Ca2+-activated chloride channel protein 2 | CLCA4 | Chloride channel activity | Cell membrane, Secreted |
| 10,31 | Human Nucleoside Diphosphate Kinase B (Nm23) | NME2 | ATP binding, DNA binding, metal ion binding, nucleoside diphosphate kinase activity, protein histidine kinase activity, sequence-specific DNA binding transcription factor activity | Cytoplasm, Nucleus |
| 11 | Alpha-enolase | ENO1 | Magnesium ion binding, phosphopyruvate hydratase activity, sequence-specific DNA binding transcription factor activity, transcription corepressor activity | Cytoplasm |
| 12 | Beta-enolase | ENO3 | Mg2+ binding, phosphopyruvate hydratase  activity | Cytoplasm |
| 13 | Cathepsin Z precursor | CTSZ | Cysteine-type peptidase activity | Lysosome |
| 14,18, 32 | Cofilin-1 | CFL1 | Cortical actin cytoskeleton, | Nucleus, Membrane, Cytoplasm,  Cytoskeleton |
| 15 | Elongation factor 1-alpha 1 | EEF1A1 | GTP binding, GTPase activity, translation elongation factor activity | Cytoplasm, Nucleus, |
|  | 14-3-3 protein epsilon | YWHAE | Potassium channel regulator activity | Cytoplasm, Melanosome |
| 16,33 | Nascent-polypeptide-associated complex alpha (HSD48) | HSD48 | DNA binding, TBP-class protein binding, transcription coactivator activity | Cytoplasm, Nucleus |
| 17 | Eukaryotic translation initiation factor 5A-1 isoform B | EIF5A | U6 snRNA binding, ribosome binding, translation elongation factor activity | Cytoplasm,  Nucleus, ER Membrane |
| 20 | Annexin A1 | ANXA1 | Calcium ion binding, phospholipase A2 inhibitor activity, protein binding, bridging, receptor binding, structural activity | Nucleus,  Cytoplasm,  Membrane |
| 22 | Methylthioadenosine phosphorylase | MTAP | S-methyl-5-thioadenosine phosphorylase activity | Cytoplasm, Nucleus |
| 23 | Eukaryotic translation initiation factor 3 subunit I | EIF3I | Translation initiation factor activity | Cytoplasm |
| 24 | Proteasome subunit p42 | PSMC6 | ATP binding, ATPase activity, protein binding, bridging | Cytoplasm, Nucleus |
| 25 | Isocitrate dehydrogenase [NAD] subunit alpha, mitochondrial precursor | IDH3A | NAD binding isocitrate dehydrogenase (NAD+) activity, magnesium ion binding. | Mitochondrion |
|  | Glyceraldehyde-3-phosphate dehydrogenase | GAPDH | NAD binding, NADP binding, glyceraldehyde-3-phosphate dehydrogenase (NAD+) (phosphorylating) activity, microtubule binding, peptidyl-cysteine, S-nitrosylase activity | Cytoplasm: Cytosol, Nucleus, Membrane,  Cytoskeleton |
| 27 | TAR DNA-binding protein 43 | TARDBP | Double-stranded DNA binding, mRNA 3'-UTR binding, nucleotide binding, sequence-specific DNA binding transcription factor activity | Nucleus |
| 28 | Serine/arginine-rich splicing factor 1 isoform 1 | SRSF1 | RNA binding, nucleotide binding | Cytoplasm, Nucleus speckle |
| 30 | Human Galectin-1 | LGALS1 | Galactoside binding, lactose binding, signal transducer activity | Secreted |
| 33 | Protein set | SET | DNA binding, histone binding, protein phosphatase inhibitor activity, protein phosphatase type 2A regulator activity | Cytoplasm, ER,  Nucleus |
