# Supplementary material for: A Proteomics Approach to Investigate miR-153-3p and miR-205-5p Targets in Neuroblastoma Cells
Source: PLoS One. 2015 Dec 3;10(12):e0143969. doi: 10.1371/journal.pone.0143969 (PMC4669106; doi:10.1371/journal.pone.0143969)
Supplement: S4 Table — (DOCX) [file pone.0143969.s006.docx]

**Table S4. Cellular processes and pathway analysis of the differentially regulated proteins in response to mimics and antagomirs of miR-153-3p and miR-205-5p**

| **miR-153-3p** | | **miR-205-5p** | |
| --- | --- | --- | --- |
| **Mimic** | **Antagomir** | **Mimic** | **Antagomir** |
| Citrate cycle (TCA cycle)  Base excision repair  Prion diseases  Pyruvate metabolism  Proteasome  Glycolysis  Gluconeogenesis  RNA degradation  Carbon metabolism  HIF-1 signaling pathway  Tuberculosis  Metabolic pathways | Glycolysis  Gluconeogenesis  Amino acids biosynthesis  Legionellosis  RNA degradation  Pancreatic secretion  Carbon metabolism  Pertussis  Fc gamma R-mediated phagocytosis  Oocyte meiosis  Pyrimidine metabolism  Ribosome  Lysosome  Cell cycle  HIF-1 signaling pathway  Axon guidance  Neurotrophin signaling  RNA transport  Hippo signaling pathway  Purine metabolism  Actin regulation  Viral carcinogenesis  Epstein-Barr virus infection  Olfactory transduction  PI3K-Akt signaling  Metabolic pathways | Amino acids biosynthesis  Carbon metabolism  2-Oxocarboxylic acid metabolism  Alzheimer's disease  Metabolic pathways  Citrate cycle (TCA cycle)  Cysteine and methionine metabolism  Proteasome  Glycolysis Gluconeogenesis  Pertussis  Fc gamma R-mediated phagocytosis  HIF-1 signaling pathway  Axon guidance  RNA transport  Epstein-Barr virus infection  Actin regulation | Leishmaniasis  Pertussis  Fc gamma R-mediated phagocytosis  Pyrimidine metabolism  Leukocyte transendothelial migration  Axon guidance  Spliceosome  Purine metabolism  Phagosome  Osteoclast differentiation  Herpes simplex infection  Actin regulation  Metabolic pathways |
|  |  |  |  |
|  |  |  |  |
|  |  |  |  |
|  |  |  |  |
|  |  |  |  |
|  |  |  |  |
|  |  |  |  |
|  |  |  |  |
